# Guideline-based management of acute respiratory failure and acute respiratory distress syndrome

**DOI:** 10.1186/s40560-023-00658-3

**Published:** 2023-03-10

**Authors:** Seitaro Fujishima

**Affiliations:** Center for General Medicine Education, Keio University School of Medicine, 35 Shinanomachi, Shinjyuku-Ku, Tokyo, 160-8582 Japan

**Keywords:** Guideline, Acute respiratory failure, Acute respiratory distress syndrome, Brain natriuretic peptide, Oxygenation targets, High-flow nasal cannula oxygenation, Noninvasive positive pressure ventilation, Corticosteroids, ECMO

## Abstract

Acute respiratory failure (ARF) is defined by acute and progressive hypoxemia caused by various cardiorespiratory or systemic diseases in previously healthy patients. Among ARF, acute respiratory distress syndrome (ARDS) is a serious condition with bilateral lung infiltration, which develops secondary to a variety of underlying conditions, diseases, or injuries. This review summarizes the current standard of care for ARF and ARDS based on current major guidelines in this field. When administering fluid in patients with ARF, particularly ARDS, restrictive strategies need to be considered in patients without shock or multiple organ dysfunction. Regarding oxygenation targets, avoiding excessive hyperoxemia and hypoxemia is probably a reasonable choice. As a result of the rapid spread and accumulation of evidence for high-flow nasal cannula oxygenation, it is now weakly recommended for the respiratory management of ARF in general and even for initial management of ARDS. Noninvasive positive pressure ventilation is also weakly recommended for the management of certain ARF conditions and as initial management of ARDS. Low tidal volume ventilation is now weakly recommended for all patients with ARF and strongly recommended for patients with ARDS. Limiting plateau pressure and high-level PEEP are weakly recommended for moderate-to-severe ARDS. Prone position ventilation with prolonged hours is weakly to strongly recommended for moderate-to-severe ARDS. In patients with COVID-19, ventilatory management is essentially the same as for ARF and ARDS, but awake prone positioning may be considered. In addition to standard care, treatment optimization and individualization, as well as the introduction of exploratory treatment, should be considered as appropriate. As a single pathogen, such as SARS-CoV-2, exhibits a wide variety of pathologies and lung dysfunction, ventilatory management for ARF and ARDS may be better tailored according to the respiratory physiologic status of individual patients rather than the causal or underlying diseases and conditions.

## Introduction

Acute respiratory failure (ARF) is defined as acute and progressive hypoxemia developing within hours, days, or up to a month caused by various respiratory, cardiovascular, or systemic disease in previously healthy patients. ARF is distinguished from chronic respiratory failure and acute exacerbations of underlying respiratory disease.

Among ARF, acute respiratory distress syndrome (ARDS) is a serious condition associated with bilateral lung infiltration. ARDS may develop secondary to a variety of underlying conditions, diseases, or injuries (Table 1) [1]. Neutrophil-dominant acute inflammation and diffuse alveolar damage (DAD) with the presence of hyaline membranes are observed on histological examination of lung tissues from patients with ARDS. The pathophysiology of ARDS includes an increase in pulmonary microvascular permeability with resultant pulmonary edema due to tissue injury and disruption of vascular regulatory mechanisms. ARDS was initially described as a single organ dysfunction, but is now recognized as one component of multiple organ dysfunction syndrome.

**Table 1:** Underlying diseases and injuries associated with ARDS

I. Direct injuries
Frequent
• Infectious pneumonia
• Gastric aspiration
Infrequent
• Fat embolism
• Inhalation injury (e.g., toxic gases)
• Ischemia reperfusion after lung transplantation
• Near drowning
• Radiation lung injury
• Pulmonary contusion
II. Indirect injuries
Frequent
• Non-pulmonary sepsis
• Severe trauma, severe burns
Infrequent
• Cardiopulmonary bypass
• Toxicant, drug overdose
• Acute pancreatitis
• Autoimmune diseases
• Blood transfusion

### Currently available guidelines for ARF and ARDS

To date, there are currently no guidelines which cover all aspects of ARF. However, several guidelines for airway and ventilatory management are available and are referred in the following sections. In addition, the Japanese clinical practice guidelines for management of sepsis and septic shock 2020 (J-SSCG 2020) includes several clinical questions and recommendations which can be extrapolated to ARF in general [2].

With regard to ARDS, the American Thoracic Society (ATS), European Society of Intensive Care Medicine (ESICM), and Society of Critical Care Medicine (SCCM) have published a joint guideline on mechanical ventilation in adult patients with ARDS. In addition, guidelines for ARDS have been published by the Faculty of Intensive Care Medicine (FICM) and Intensive Care Society (ICS) of United Kingdom (jointly as guidelines on the management of acute respiratory distress syndrome: FICM/ICS-ARDS-GL2018), Société de Réanimation de Langue Française (SRLF) of France (management of acute respiratory distress syndrome: SRLF-ARDS-GL2019), Scandinavian Society of Anaesthesiology and Intensive Care Medicine (SSAI; Scandinavian clinical practice guideline on mechanical ventilation in adults with the acute respiratory distress syndrome: SSAI-ARDS-GL2016), and Korean Society of Critical Care Medicine (KSCCM) and Korean Academy of Tuberculosis and Lung Diseases (KATRD) of South Korea (jointly as the clinical practice guideline of acute respiratory distress syndrome: KSCCM/KATRD-ARDS-GL2016) [3–8]. In Japan, an initial guideline was developed in 2005 by the Japanese Respiratory Society (JRS), with the latest version jointly published in 2022 by the JRS, Japanese Society of Intensive Care Medicine (JSICM), and Japanese Society of Respiratory Care Medicine (JSRCM) as the ARDS clinical practice guideline 2021 (Japanese ARDS-GL2021) [9]. In addition, the Surviving Sepsis Campaign Guidelines (international guidelines for management of sepsis and septic shock 2021; SSCG2021) also include clinical questions regarding ventilatory management.

Since early 2020, novel coronavirus-induced disease 2019 (COVID-19) has become a major cause of ARF and ARDS. The large number of cases caused by a single microorganism is unprecedented in modern times. The above-mentioned guidelines are generally applicable to ARF and ARDS caused by COVID-19. However, specific guidelines for the management of COVID-19 should also be consulted as many international and regional guidelines for COVID-19 have now been published [10] based on evidence specific to COVID-19.

### Diagnosing ARF and ARDS

ARF is typically diagnosed according to a PaO_2_ ≤ 60 Torr at room air or PaO_2_/FIO_2_ ratio ≤ 300. ARF can be caused by a range of lung, heart, or other systemic diseases and conditions. American College of Physicians has developed a guideline for the appropriate use of point-of-care ultrasonography in patients with acute dyspnea, and weakly recommends its use in addition to the standard diagnostic pathway when there is diagnostic uncertainty [11].

The clinical diagnosis of ARDS is currently based on the Berlin definition: (1) PaO_2_/FIO_2_ ratio ≤ 300 under positive end-expiratory pressure (PEEP)/continuous positive airway pressure (CPAP) ≥ 5 cmHO_2_; (2) acute onset within a week; (3) bilateral shadows in the lung fields, and (4) respiratory failure that cannot be explained by cardiac failure or excess fluid alone [12]. Recently, high-flow nasal cannula oxygenation (HFNC, also called high-flow nasal oxygen therapy: HFNO or nasal high flow therapy: NHFT) and noninvasive positive pressure ventilation (NPPV, also called NIV) have become widely used, with an SpO_2_/FIO_2_ ratio ≤ 315 irrespective of PEEP proposed as an alternative criterion of ARDS [13].

Fluid balance assessments, levels of plasma brain natriuretic peptide (BNP) or serum NT-proBNP, and echocardiographic evaluation are clinically used in differentiating ARDS from hydrostatic pulmonary edema. In JRS/JSICM/JSRCM-GL2021, a systematic review reported a sensitivity of 0.77 and specificity of 0.62 for a cutoff value of 400–500 pg/mL for BNP, sensitivity of 0.50 and specificity of 0.82 for a cutoff value of 1000 pg/mL, and sensitivity of 0.71 and specificity of 0.89 for a cutoff value of 4000 pg/mL for NT-proBNP when differentiating ARDS from hydrostatic pulmonary edema. According to these results, the use of serum BNP or NT-proBNP levels is weakly recommended [9]. In patients with severe ARDS, measurement of extravascular lung water using transpulmonary thermodilution should be considered. Measurement of pulmonary artery wedge pressure by invasive right heart catheterization is now rarely performed.

After clinical exclusion of hydrostatic pulmonary edema, the diagnosis of ARDS is made according to the aforementioned diagnostic criteria. However, it is still necessary to rule out ARDS mimics, particularly those with established treatments (Table 2) [1, 14]. Bronchoalveolar lavage is particularly useful in differentiating various respiratory infections, acute eosinophilic pneumonia, cryptogenic organizing pneumonia, interstitial pneumonia, hypersensitivity pneumonitis, alveolar hemorrhage, and drug-induced lung injury.

**Table 2:** Diseases and conditions that require differentiation from ARDS

1. Hydrostatic pulmonary edema
2. Pneumonia: bacterial, viral, Pneumocystis, fungal, tuberculous, etc.
3. Diffuse alveolar hemorrhage (DAH), pulmonary capillaritis
4. Acute eosinophilic pneumonia (AEP)
5. Cryptogenic organizing pneumonia (COP)
6. Acute exacerbation of chronic interstitial pneumonia (IP)/idiopathic pulmonary fibrosis (IPF)
7. Acute interstitial pneumonia (AIP)
8. Hypersensitivity pneumonitis (HP)
9. Alveolar proteinosis
10 Malignant tumors (lymphoma, metastatic cancer), lymphangitis carcinomatosa
11. Drug-induced lung injury
12. Other noncardiogenic causes of pulmonary edema: re-expansion, neurogenic, high altitude, negative pressure, etc.

### Management of ARF and ARDS

In this section, the current standard approach to the management of ARF and ARDS is presented based on recent guidelines. Recommendations for ARF are given in SSCG2021, J-SSCG2020, and SRLF-GL2019, and are summarized in Table 3. For ARDS, recommendations for ventilatory management are summarized in Table 4, and those for adjunctive therapies are presented in Table 5. Key topics in the above-mentioned guidelines are discussed below with reference to recent evidence.

**Table 3:** Recommendations for acute respiratory failure in major guidelines

	SSCG 2021	J-SSCG 2020	SRLF-GL 2019
Ventilatory management			
Lower PaO_2_/SpO_2_ target	–	B	
HFNC	B	B	
NPPV	–		
Lung protective strategy			
Low tidal volume	B	B	C
Low plateau pressure			
High PEEP		D	
Weening protocolization		B	
Post-extubation NPPV/HFNC		B	

A, strongly recommended; B, weakly/conditionally recommended; C, expert opinion/research recommendation; D, weakly/conditionally not recommended; E, strongly not recommended; -, no recommendation

*HFNC* high-flow nasal cannula oxygenation, *NPPV* noninvasive positive pressure ventilation

**Table 4:** Recommendations for acute respiratory distress syndrome in major guidelines: (1) ventilatory management

	JRS/JSICM/JSRCM-GL2021	SSCG 2021	SRLF-GL 2019	FICM/ICS- GL 2018	ATS/ESICM/SCCM-GL2017	SSAI-ARDS-GL2016	KSCCM/KATRD-ARDS-GL2016
Lower SpO_2_ (PaO_2_) target	D for excess control	-				–	
HFNC	B						
NPPV	B					–	
Lung protective ventilation							
Low tidal volume	A	A	A	A	A	A	A
Low plateau pressure	B	A Severe	A		A	A	–
High level PEEP	B	A: Moderate–severe	A	B: P/F ≤ 200	C: Moderate–severe	B	B
Recruitment maneuver	D	B: Moderate–severe traditional	E: routine use		C	B	B
Prone position	B: Long hours	A: Moderate–severe, ≥ 12 h	A: P/F < 150, ≥ 16 h	A: P/F < 150, ≥ 12 h	A: ≥ 12 h	B	A
High-frequency oscillatory ventilation (HFOV)	D		E	E	E	E	E
Limited muscle relaxants use	B: Moderate–severe	B: Moderate–severe, intermittent use	A: P/F < 150, ≤ 48 h	B: P/F ≤ 150, ≤ 48 h		B	B
Weening protocolization	B						
Early tracheotomy	B						D

A, strongly recommended; B, weakly/conditionally recommended; C, expert opinion/research recommendation; D, weakly/conditionally not recommended; E, strongly not recommended; –, no recommendation

**Table 5:** Recommendations for acute respiratory distress syndrome in major guidelines: (2) adjunctive therapies

	JRS/JSICM/JSRCM-GL2021	SSCG2021	SRLF-GL 2019	FICM/ICS- GL 2018	SSAI-ARDS-GL2016	KSCCM/KATRD-ARDS-GL2016	CIRCI-GL2017
Restrictive fluid management	B			B	B		
Pharmacotherapy							
Corticosteroids	A (1–2 mg/kg)			C	E	D	B
Neutrophil elastase inhibitor	D						
Beta_2_-agonists	E				E		
NO inhalation	D		C	D	E	E	
Thrombomodulin	–						
ECMO	B: Severe	B: V-V, severe	A: V-V	B: Severe		B	
Early rehabilitation	B: ≤ 72 h						
No or light sedation	B					A	
Omega-3 fatty acids enteral nutrition	B						

A, strongly recommended; B, weakly/conditionally recommended; C, expert opinion/research recommendation; D, weakly/conditionally not recommended; E, strongly not recommended; -, no recommendation

*ECMO* extracorporeal membrane oxygenation, *V-V* veno-venous

### Oxygenation targets

The traditional treatment strategy regarding oxygenation in ARF is to maintain adequate oxygenation to avoid the risk of hypoxemia. On the other hand, it has been customary to aim for an FIO_2_ ≤ 60% to avoid hyperoxic lung injury in ventilated patients. However, a recent systematic review and cohort study reported a positive association between hyperoxemia and poor survival. As a result, optimal oxygenation targets have again become a topic of discussion [15, 16]. After 2016, six RCTs comparing groups with lower and higher oxygen targets were published, with none reporting a significant difference in primary outcomes between the two groups [17–21]. In these studies, the actual difference between study groups was 15–28 mmHg in PaO_2_ or 1–4% in SaO_2_, and PaO_2_ was maintained between 70 and 110 mmHg in both groups in all studies. These situations have resulted in inconsistent recommendations between SSCG2021, J-SCG2020, and JRS/JSICM/JSRCM-GL2021 as shown in Tables 3 and 4. As a recent network meta-analysis demonstrated decreased survival in patients with a PaO_2_ target of 55–75 mmHg and patients with a PaO_2_ ≥ 150 mmHg, it seems appropriate to follow the traditional oxygenation strategy that avoids excess hypoxemia and hyperoxemia [22]. In patients with acute exacerbation of chronic obstructive pulmonary disease (COPD), an SaO_2_ of 88% to 92% is considered an adequate oxygenation target, as suggested by a recent observational study [23].

### Ventilatory management

In ARF, the choice between the use of nasal cannula, HFNC, NPPV, or invasive positive pressure ventilation (IPPV) is based on the presence of underlying disease and severity of hypoxemia. In the HFNC guidelines by the American College of Physicians, HFNC was weakly recommended for ARF over NPPV due to a systematic review reporting that HFNC for ARF is associated with lower mortality and a lower intubation rate compared to NPPV [24]. For patients with ARF post-extubation, a separate systematic review suggested that HFNC may reduce the reintubation rate and improve patient comfort compared with conventional oxygen therapy, and thus was also weakly recommended. The European Respiratory Society (ERS)/ATS guidelines recommend bilevel positive airway pressure (bilevel-PAP) for patients with acute exacerbation of COPD accompanied by acute hypercarbia, CPAP for cardiogenic pulmonary edema, and NPPV for post-operative setting and early ARF in immunosuppressed patients [25]. Regarding ARDS, IPPV has been the gold standard; however, HFNC and NPPV are weakly recommended as alternative options to initial management in JRS/JSICM/JSRCM-GL2021.

The benefit of low tidal volume ventilation with IPPV has been demonstrated not only in ARDS, but also in ARF. Low tidal volume ventilation is weakly recommended for ARF in SSCG2021 and SRLF-GL2019, and strongly recommended for ARDS in JRS/JSICM/JSRCM-GL2021, SSCG2021, SRLF-GL2019 and FICM/ICS-GL2018. In J-SSCG2020, lung protective ventilation is weakly recommended for ARF.

Limiting plateau pressure and high-level PEEP is recommended weakly to strongly in all guidelines, although the most recent Cochrane analysis did not find a survival benefit for high-level PEEP [26]. Prone position ventilation with prolonged hours is weakly to strongly recommended for moderate-to-severe ARDS in all guidelines. Regarding recruitment maneuvers, JRS/JSICM/JSRCM-GL2021 recommends against their routine use while the SSCG 2021 weakly recommends the traditional recruitment maneuver of applying an airway pressure of 30–40 cm H_2_O for 30–40 s [9, 27, 28]. Early and limited use of muscle relaxants are weakly to strongly recommended for patients with moderate to severe ARDS. There are weak-to-strong recommendations against the use of high-frequency oscillatory ventilation (HFOV).

### Fluid management

There are currently no standardized guidelines for fluid management in ARF; however, daily fluid balance assessments are fundamentally important in reducing the risk of iatrogenic pulmonary edema. Even mild fluid overload may worsen pulmonary edema and thereby exacerbate hypoxemia in patients with ARDS due to an increase in pulmonary microvascular permeability. A recent systematic review reported that restrictive fluid management improves oxygenation and prolongs ventilator-free days, but does not improve mortality in patients with sepsis or ARDS [29]. Based on this evidence, the JRS/JSICM/JSRCM-GL2021 and FICM/ICS-GL 2018 weakly recommend restrictive fluid management [4, 9].

On the other hand, stabilization of vital signs with fluid resuscitation is essential in sepsis and septic shock, which is a major cause of ARDS. Accordingly, an appropriate fluid management strategy should be selected in patients with ARDS depending on the presence of other organ dysfunction or hemodynamic shock [30]. In the most recent RCT for patients with septic shock, a trend toward increased survival was observed in a subgroup with respiratory support, although restrictive fluid management did not show overall survival benefit [31], supporting the use of the above strategy. In severe cases, echocardiography and measurement of central venous pressure should be performed to monitor fluid responses and inform fluid administration.

### Pharmacotherapy

In ARF, pharmacotherapy should be focused on the underlying disease or diseases that are causing hypoxemia. For ARDS, corticosteroids are often administered worldwide including Japan [32]. However, the results of RCTs for pharmacological treatment of ARDS have been mixed due to diversity in the causes and severity of ARDS and the effects of the type, timing of administration, dosage, and duration of administration of corticosteroids. Accordingly, corticosteroid administration is considered both a standard and exploratory treatment for ARDS. The latest RCT “DEXA-ARDS” included 277 patients with a PaO_2_/FIO_2_ ≤ 200 mmHg under PEEP ≥ 10 cmHO_2_ and a FIO_2_ ≥ 0.5 at 17 Spanish intensive care units. Patients in the dexamethasone group were treated with 20 mg intravenous dexamethasone (methylprednisolone equivalent 100–120 mg) daily for five days and 10 mg for additional five days [33]. A recent systematic review that included 18 RCTs also demonstrated a net survival benefit for corticosteroids in patients with ARDS of any cause [34]. Based on these findings, it can be suggested that although older versions such as SSAI-ARDS-GL2016 and KSCCM/KATRD-ARDS-GL2016 are against the use of corticosteroids, their use is weakly to strongly recommended in the more recent JRS/JSICM/JSRCM-GL2021 and FICM/ICS-GL2018. Guidelines for the diagnosis and management of critical illness-related corticosteroid insufficiency (CIRCI) include ARDS and weakly recommend the use of corticosteroids [35].

A specific neutrophil elastase inhibitor, sivelestat, was developed and approved for the treatment of acute lung injury associated with systemic inflammatory response syndrome in Japan. In the Japanese ARDS guidelines 2016, a systematic review was performed including data from the Japanese phase III trial and the international phase III STRIVE study, with no difference in survival or ventilator-free days observed [1]. Based on these findings, the latest JRS/JSICM/JSRCM-GL2021 also weakly recommends against the routine use of sivelestat.

In situations where respiratory infections cannot be ruled out, the use of broad-spectrum antibiotic regimens including a macrolide or new quinolone is often considered. Antimicrobial therapy against methicillin-resistant *Staphylococcus aureus*, *Pneumocystis jirovecii*, fungi, *Mycobacterium tuberculosis*, viruses, and SARS-CoV-2 may also be considered as appropriate.

### Extracorporeal membrane oxygenation

The benefit of extracorporeal membrane oxygenation (ECMO) has been clarified in recent studies, with ECMO now weakly recommended for severe ARDS in most guidelines. The systematic review of the newest JRS/JSICM/JSRCM-GL2021 included two RCTs (CESAR and EOLIA studies) and found a significant decrease in 60-day and 90-day mortalities but no increase in the incidence of stroke [9].

However, it is important to recognize and follow the accepted indications and contraindications for ECMO to obtain improved implementation results. In the latest ELSO guidelines, common indications for veno-venous ECMO are: (1) hypoxemic respiratory failure (PaO_2_/FiO_2_ < 80 mmHg) after optimal medical management including, in the absence of contraindications, a trial of prone positioning; (2) hypercapnic respiratory failure (pH < 7.25) despite optimal conventional mechanical ventilation (respiratory rate 35 breaths per minute and plateau pressure [Pplat] ≤ 30 cm H_2_O); and (3) ventilatory support as a bridge to lung transplantation or primary graft dysfunction following lung transplantation [36]. Central nervous system hemorrhage, significant central nervous system injury, irreversible and incapacitating central nervous system pathology, systemic bleeding, contraindications to anticoagulation, immunosuppression, older age (increasing risk of death with increasing age but no threshold is established), and mechanical ventilation for more than seven days with a Pplat > 30 cm H_2_O and an FiO_2_ > 90% are listed as relative contraindications to ECMO.

### COVID-19

A certain proportion of patients with COVID-19 develop ARF and ARDS depending on patient age, comorbidities, immune status, and SARS-CoV-2 virus genotype among other factors. Although there are rare cases with a rapidly progressive course, the progression of the disease is typically slow and the number of days from the onset of symptoms to the start of artificial ventilation is as high as 3–4 days for the original variant of SARS-CoV-2 [37]. The rate of severe illness is lower in Omicron variants of SARS-CoV-2 compared to Delta variants; however, the mortality of the patients once admitted to ICU does not differ between Omicron and Delta variants [38].

In the chaotic early stages of the COVID-19 pandemic, a specific phenotype of COVID-19-induced ARDS with higher lung compliance was proposed and discussed [39]. However, after the accumulation of numerous cases worldwide over more than two years, a recent systematic review did not find evidence of a specific phenotype of ARDS related to COVID-19 [40]. These findings indicate that the management of ARF and ARDS in patients with COVID-19 should be the same as for other causes. However, parameters of mechanical ventilation, including PEEP, should be individualized based on the ventilatory and systemic condition of individual patients [41]. Pharmacological therapies, including corticosteroids, should be administered according to the guidelines and statements specific to COVID-19.

In addition to standard ventilatory management, the benefits of awake prone positioning for non-intubated patients have been posited and examined. Although the results of RCTs are conflicting, a recent systematic review demonstrated a reduced risk of endotracheal intubation with awake prone positioning [42].

The criteria for the introduction of ECMO and the survival rate in COVID-19 are similar to those in other diseases; however, the duration of ECMO use tends to be longer in patients with COVID-19 [43]. In a recent systematic review, increased mortality was reported to be associated with older age, male sex, chronic lung disease, longer duration of symptoms, longer duration of invasive mechanical ventilation, higher PaCO_2_, higher driving pressure, and less previous experience with ECMO [44].

## Concluding remarks

ARF and ARDS develop secondary to a wide variety of diseases and conditions, and the mechanisms of hypoxemia are varied. This review summarized the current standard of care for ARF and ARDS based on major guidelines in this field. As has been repeatedly mentioned, “standard” care needs to be continually updated considering new evidence. In addition to standard care, treatment optimization and individualization as well as the introduction of exploratory treatment should be considered appropriate. In light of the fact that even a single pathogen, such as SARS-CoV-2, exhibits a wide variety of pathologies and lung dysfunction, ventilatory management for ARF and ARDS may be suitably tailored according to the respiratory physiologic status of individual patients rather than the causal or underlying diseases and conditions.

## Data Availability

Not applicable.

## Abbreviations

ARDS: Acute respiratory distress syndrome
ARF: Acute respiratory failure
ATS: American Thoracic Society
Bilevel-PAP: Bilevel positive airway pressure
BNP: Brain natriuretic peptide
CIRCI: Critical illness-related corticosteroid insufficiency
COPD: Chronic obstructive pulmonary disease
COVID-19: Coronavirus-induced disease 2019
CPAP: Continuous positive airway pressure
DAD: Diffuse alveolar damage
ECMO: Extracorporeal membrane oxygenation
ELSO: Extracorporeal Life Support Organization
ESICM: European Society of Intensive Care Medicine
FICM: Faculty of Intensive Care Medicine
HFNC: High-flow nasal cannula
HFNO: High-flow nasal oxygen therapy
HFOV: High-frequency oscillatory ventilation
ICS: Intensive Care Society
IPPV: Invasive positive pressure ventilation
JRS: Japanese Respiratory Society
J-SCG2020: Japanese clinical practice guidelines for management of sepsis and septic shock 2020
JSICM: Japanese Society of Intensive Care Medicine
JSRCM: Japanese Society of Respiratory Care Medicine
KATRD: Korean Academy of Tuberculosis and Lung Diseases
KSCCM: Korean Society of Critical Care Medicine
NIV: Noninvasive ventilation
NHFT: Nasal high-flow therapy
NPPV: Noninvasive positive pressure ventilation
PEEP: Positive end-expiratory pressure
SARS-CoV-2: Severe acute respiratory syndrome coronavirus 2
SCCM: Society of Critical Care Medicine
SRLF: Société de Réanimation de Langue Française
SSAI: Scandinavian Society of Anaesthesiology and Intensive Care Medicine
qaSSCG: Surviving Sepsis Campaign Guidelines
